# The Influence of Metakaolinite on the Development of Thermal Cracks in a Cement Matrix

**DOI:** 10.3390/ma11040520

**Published:** 2018-03-29

**Authors:** Maciej Szeląg

**Affiliations:** Faculty of Civil Engineering and Architecture, Lublin University of Technology, 40 Nadbystrzycka Str., 20-618 Lublin, Poland; maciej.szelag@pollub.pl; Tel.: +48-81-538-4428

**Keywords:** cluster cracks, clusters, cement paste, metakaolinite, image analysis, dispersion system

## Abstract

In the paper the cluster cracks of cement paste that has been modified with metakaolinite was analyzed. The samples were loaded with an elevated temperature based on a thermal shock. To describe the crack structure, three stereological parameters were proposed to measure: (i) the cluster average area ($\bar{A}$); (ii) the cluster average perimeter ($\bar{L}$); and (iii) the crack average width ($\bar{I}$). The computer image analysis was implemented in the study, and 4 series of samples were subjected to the examination. In two series, metakaolinite was used as a substitute for 10% of a cement’s mass. An assessment of the basic physico-mechanical characteristics of the cement matrix was also carried out. The structure of the cement paste was considered as a highly concentrated dispersion system, in which the interactions between the cement’s grains at the initial stage of the structure self-assembly affect the crack characteristics. The study has been supplemented with microstructural investigations using a scanning electron microscope and an X-ray microanalyzer. The conducted research indicated the direction of changes in the geometrical characteristics of thermal cracks if the technological variables of the material are subjected to modification. It was also confirmed that the cluster structures have fractal character and can be analyzed and observed on many levels of a structural heterogeneity.

## 1. Introduction

Volumetric deformation of a cementitious material is one of the effects of a thermal stress impact on its structure. As a result, the composite may crack, and cracks through propagation combine or intersect, which leads to a creation of the specific crack structure defined as the thermal cracks—the cluster cracks—or the map cracking [1,2,3]. The analysis of this crack structure seems to be important from a technological point of view, because the cracking of the cement matrix weakens the material’s structure and increases the risk of penetration of harmful substances into the composite. This causes a progressive degradation of the material, and in the case of a reinforced concrete, reinforcement corrosion may occur. On the other hand, the analysis of the thermal cracks poses methodological challenges. Image analysis tools that have already found use in the concrete technology for crack analysis are one of the solutions. However, this is a local analysis of cracks and parameters of individual cracks are measured, e.g., the opening width, crack length, etc. [4,5,6,7,8,9,10,11,12]. There is a lack of research work, in which the cluster crack structure is analyzed in a global way, and the results obtained are related to material properties.

The microstructure of cement paste can be described as a dispersion system [1,13,14,15,16]. A dispersive phase is cement grains decomposed in the continuous phase (water). The structure of a dense suspension, which is a cement paste immediately after mixing cement with water, depends on many factors, e.g., the concentration of the dispersive phase (*w/c*), its fineness, and the forces acting at the interface between the phases. In the description of the separation boundary, the following quantities are used: the surface formation enthalpy, the Gibbs free energy, and the capillary forces that are responsible for the cement grains flocculation [1,13,17,18]. The structural aggregates created because of flocculation form the clusters of various sizes and different concentrations of the dispersive phase. The clusters occur at all levels of the material’s structural heterogeneity and are subjected to the process of the self-assembly. Their main feature is the fractal nature, which allows observation of them at each of the structure’s level [1,19,20,21,22,23,24]. The key elements in this description are the separation surfaces between clusters that appear in the form of technological cracks, created at the initial stage of formation of the cement matrix structure. External influences cause volumetric deformations of cement paste as well as individual clusters. The result is the transformation of the technological cracks into microcracks, and then into macrocracks, which are visible on the material’s surface [13,20,25].

The aim of the research was to determine the effect of the pozzolanic component in the form of metakaolinite (MT) on the geometric characteristics of thermal cracks of cement matrix. The cluster crack structure was described quantitatively using stereological parameters, which were measured based on the analysis of the scanned surface of the cracked cement paste. In addition, the basic mechanical and physical characteristics of the material were determined. An analysis of the local microstructure of cement matrix using the scanning electron microscope (SEM) and the X-ray microanalyzer (EDS) was also carried out. This allowed observation of the cluster microcracks at the lower level of the structure.

## 2. Materials and Methodology

### 2.1. Materials Used, Samples Maturation and Thermal Load

The tests were carried out on 4 series of cement paste samples. For each series, samples were made with 3 water/binder ratios; *w/b* = 0.4, 0.5, and 0.6. The application of the *w/b* ratio resulted from the fact that MT was used as a cement substitute. The following series of cement pastes were made:

- C42—100% CEM I 42.5R and water,
- C42MT—90 CEM I 42.5R, 10% MT, and water,
- C52—100% CEM I 52.5R and water,
- C52MT—90 CEM I 52.5R, 10% MT, and water.

Portland cements of two different classes—CEM I 42.5 R and CEM I 52.5 R were used (CEMEX, Chełm, Poland). Cements are characterized by a very similar chemical composition, but they differ in the degree of grain refinement, which is represented by the specific surface area—Table 1. A similar oxide composition translates directly into a similar mineral composition, which was calculated using the Bogue’s formula [26].

Samples were made in the form of bars measuring 40 × 40 × 160 mm. The cement paste was laid in molds, in two layers, successively compacted using a standard shaker—according to EN 196-1 [27]. All tests planned were performed after the maturation period, i.e., 28 days at a temperature of about 20 °C and an average relative humidity of 50%.

After the maturation period, the samples were exposed to an elevated temperature. The furnace was pre-heated to a temperature of 250 °C, then the samples were put into it for a period of 4 h. After heating, the samples were removed from the furnace and cooled down by a natural temperature drop in laboratory conditions (about 20 °C). The method of thermal loading corresponds to a thermal shock. The result of the thermal load was the appearance of the thermal cracks on the sample’s surface. These cracks were created mainly as a result of the material’s volumetric deformation—thermal expansion in the heating phase, shrinkage associated with the evaporation of water, and shrinkage in the cooling phase. In the temperature range up to 250 °C, the cement matrix is chemically and thermally stable. The main effect of heating at this temperature is the intensive evaporation of free water [28].

The pressure of water vapor formed in the pores and capillaries of the cement matrix causes the propagation and transformation of the technological cracks into the macrocracks, which are visible on the sample’s surface [13]. In this way, the defects of the cement matrix structure were extracted, without excessive deterioration of the mechanical properties. The thermal loading method was determined on the basis of the tests carried out at different temperatures and with different duration of load, based on previous author’s studies [13,25,29,30,31,32,33].

### 2.2. Examination of the Selected Physical and Mechanical Features

The tensile strength (*f_cf_*) has been tested in a three-point bending scheme in accordance with PN-EN 12390-5 [34]. The strength was tested on samples after 28 days of maturation (*f_cf(R)_*), and on samples subjected to the thermal action (*f_cf(T)_*). The results presented for each series and the *w/b* ratio are the arithmetic mean obtained from 6 samples. The sample halves created after the test were used to determine the compressive strength.

The compressive strength test (*f_c_*) was carried out in accordance with PN-EN 12390-3 [35]. As in the case of *f_cf_*, the test was carried out on samples after 28 days of maturation (*f_c(R)_*), and on samples after thermal loading (*f_c(T)_*). The results obtained were presented in the form of the mean value of 12 measurements.

The apparent density (*D*) of the hardened cement matrix was determined in accordance with PN-EN 12390-7 [36]. The parameter was determined after 28 days of maturation (*D_(R)_*), as well as for samples loaded with the elevated temperature (*D_(T)_*). The results are the arithmetic mean of 6 samples.

### 2.3. Image Analysis

**Figure 1:**
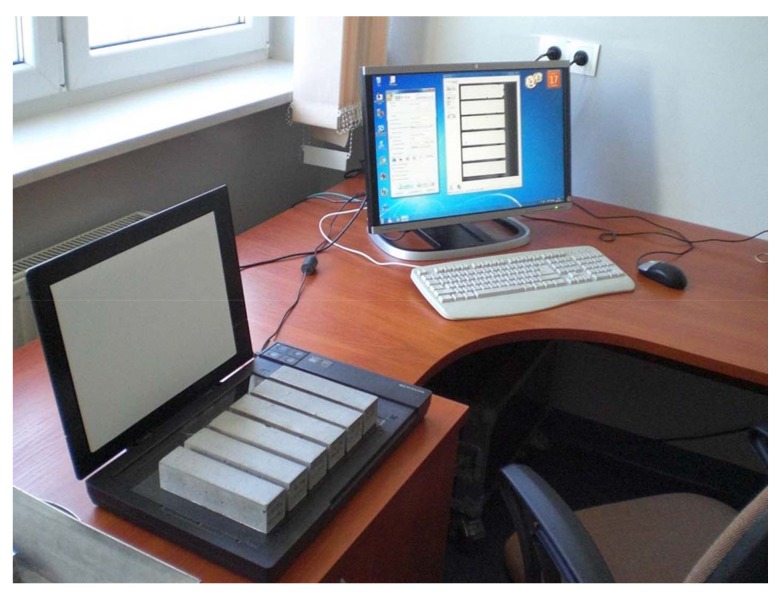
Test stand for scanning the surface of cement paste.

The cracked surface of the cement paste samples was scanned using an optical scanner (Epson, Suwa, Japan). The scanning was performed on an 8-bit gray scale, at a resolution of 2400 DPI, and with a sharpening mask. The test stand is shown in Figure 1. The purpose of scanning at such a high resolution was to obtain the most detailed image of the specimen’s surface. With a resolution of 2400 DPI per 1 mm of a surface scanned, there is 94.5 pixels. This allowed for a positive identification of a crack with a width of 2–3 pixels, i.e., approximately 0.021 mm. Cracks with such a width are not visible to the naked eye, and their detection should be considered as a good result, especially when no magnifying optics were used during scanning.

To quantify the surface structure of the cluster cracks, the use and measurement of three stereological parameters was proposed, among the local (statistical), metric parameters:

- $\bar{A}$—the cluster average area—the parameter of R^(2)^ space,
- $\bar{L}$—the cluster average perimeter—the parameter of R^(2)^ space,
- $\bar{I}$—the crack average width—the parameter of R^(1)^ space.

**Figure 2:**
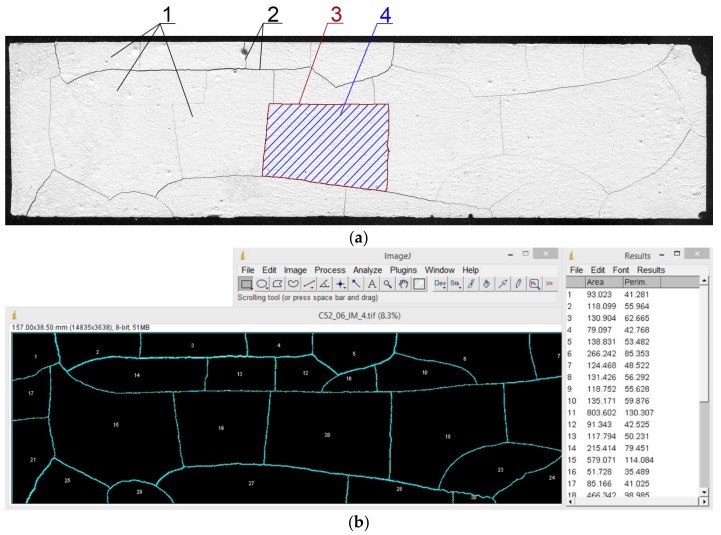

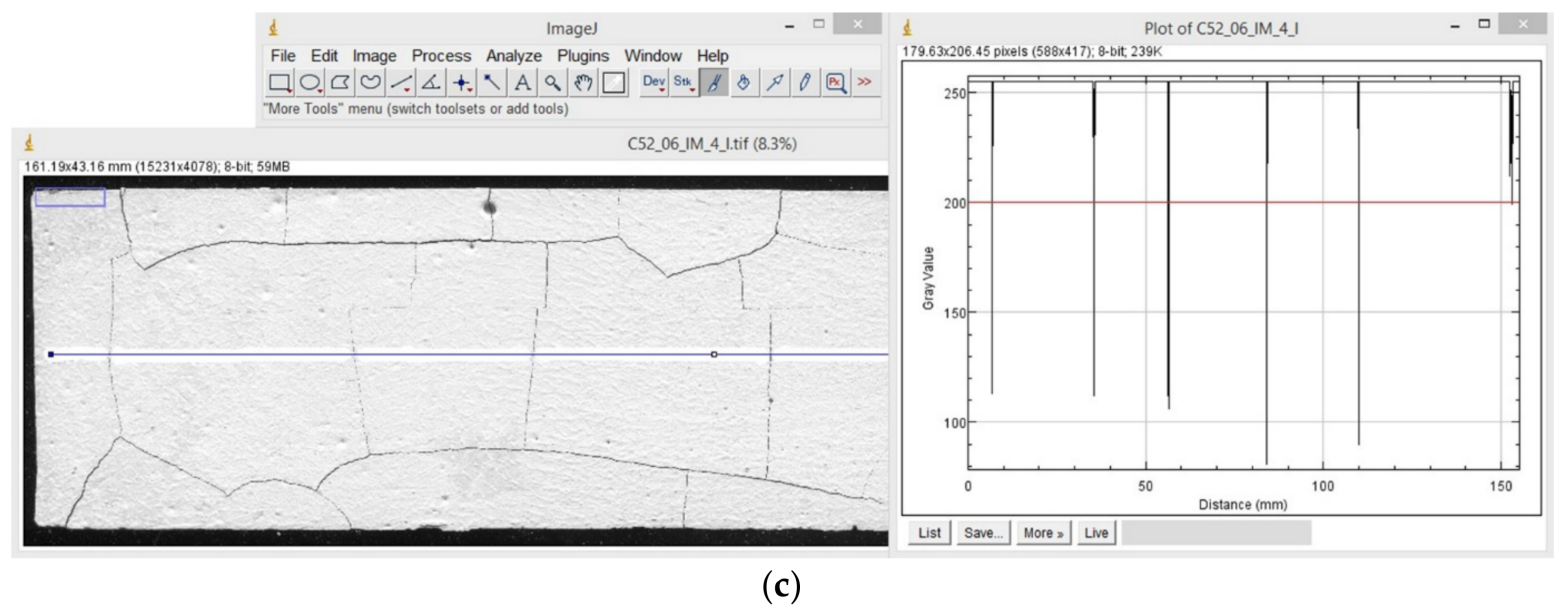
Scanned surface of the cement paste: (**a**) the primary image; (**b**) the image after processing and the $\bar{A}$ and $\bar{L}$ measurements; (**c**) the image after processing and the $\bar{I}$ measurements; 1—the clusters; 2—cracks forming the separation surface between clusters; 3—the cluster perimeter; 4—the cluster area.

Image analysis (graphic processing and measurement of the stereological parameters) was carried out using the ImageJ software (v.1.51j8, National Institutes of Health, Bethesda, Rockville, MD, USA). The measuring surface has been limited to the area of 157 mm × 38.5 mm. This was conditioned by the determination of a constant surface area for each sample because due to the thermal load, the cement paste was subjected to the volumetric deformations. Contrast and sharpness filters were implemented in the area analyzed, and then, for the measurements, it was transformed into a binary image. Using the “analyze particles” module, the $\bar{A}$ and $\bar{L}$ were measured. The “plot profile” module was used to measure $\bar{I}$. The measurement procedure has been described in detail in [25]. An exemplary image of a sample before and after image analysis is shown in Figure 2.

This characteristic network of cracks is also visible at the sample’s fracture created after the *f_cf_* test. Therefore, it is assumed that cluster cracks concern the entire volume of the material, not just its surface. This property causes that the results obtained from the image analysis of the surface cracks structure can be related to the self-assembly process of the cement matrix structure. The occurrence and development of the separation surfaces between clusters can be described by thermodynamic quantities and explained on the basis of the interactions analysis that occur in the highly-concentrated dispersion system, which is the cement matrix at the initial stage of shaping its internal structure [1,2,14,16,20].

### 2.4. SEM and EDS

Analysis of the local microstructure of the modified cement matrix was carried out based on images obtained from SEM (FEI, Hillsboro, OR, USA). The samples for the analysis were broken off from the inner part of the sample. To obtain a conductive layer, the samples were sputtered with carbon. The test was carried out after 28 days of maturation, both on reference samples and those subjected to the thermal action. The photos were taken in a high vacuum mode. On the same samples, the EDS analysis (Energy Dispersive X-ray Spectroscopy, FEI) was carried out, which allowed for the identification of the local chemical composition of the material.

## 3. Results and Discussion

### 3.1. Mechano-Physical Properties of the Cement Pastes

**Figure 3:**
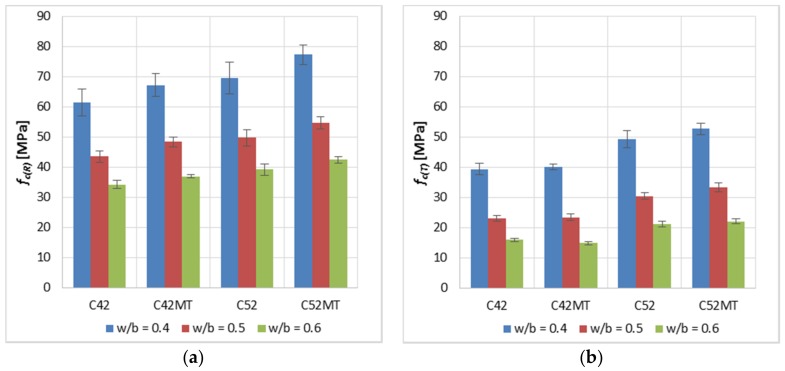
Average compressive strength *f_c_* of the modified cement pastes (error bars mean standard deviations): (**a**) reference samples; (**b**) samples after thermal load.

Analyzing the *f_c(R)_* and *f_c(T)_* results (Figure 3), it was noticed, as predicted, that higher values of the mechanical properties were achieved by samples made of a higher-class cement, i.e., CEM I 52.5R. The C52 and C52MT cement pastes reached higher values than samples made of CEM I 42.5R by an average of 14.1% and 33.4%—for *f_c(R)_* and *f_c(T)_* respectively. This indicates that the cement matrix made of the higher-class cement is more resistant to the thermal shock.

The effect of MT on *f_c_* is particularly noticeable in the case of the reference specimens. The C42MT and C52MT series obtained higher *f_c(R)_* values by an average of 9.8% compared to C42 and C52 samples. However, after exposure to the elevated temperature, this difference decreased twice and amounted to 3.9%. The MT reacts with the calcium hydroxide forming mainly the CSH, C_4_AH_13_ and gehlenite phases. The presence of MT in the cement matrix causes its sealing by filling the free spaces between the cement grains and reducing the porosity of cement paste (similar effect as in the case of microsilica). These mechanisms combined with a reduction in the CH amount cause an increase in the compressive strength.

**Figure 4:**
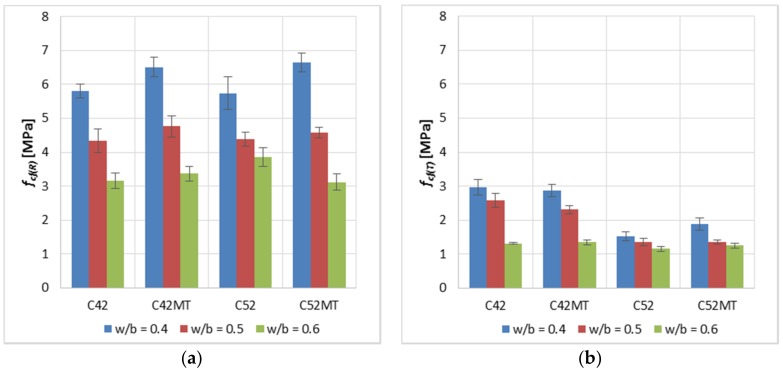
Average tensile strength *f_cf_* of the modified cement pastes (error bars mean standard deviations): (**a**) reference samples; (**b**) samples after thermal load.

The effect of the cement’s class on *f_cf(R)_* (Figure 4) was negligible; the average difference between the values was 1.4%, in favor of cement pastes made of CEM I 52.5R. However, in the case of *f_cf(T)_*, the C52 and C52MT series obtained lower values by an average of 36.5% from the C42 and C42MT. This is due to the brittleness of the cement matrix; in general, a cement matrix with a higher *f_c_* is more brittle [37,38,39], which makes the material absorb relatively little energy before the crack develops. This leads to the ultimate limit state being exceeded in the aspect of the cement matrix stretching.

A positive effect of MT on *f_cf(R)_* was observed; the C42MT and C52MT series obtain higher values by an average of 6.2% than samples of C42 and C52 series. However, after exposure to the elevated temperature, the effect of MT on *f_cf(T)_* was negligible; the average difference in values was 0.9%.

**Figure 5:**
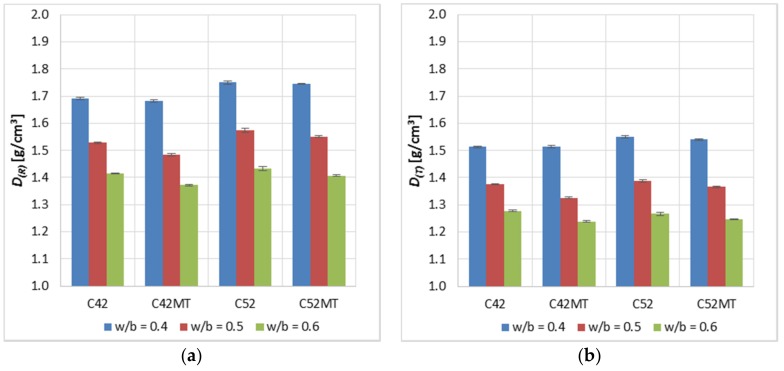
Average bulk density *D* of the modified cement pastes (error bars mean standard deviations): (**a**) reference samples; (**b**) samples after thermal load.

The apparent density of cement pastes (Figure 5) made of CEM I 52.5R was on average higher by 3.1% and 1.4% compared to the CEM I 42.5R samples—for *D_(R)_* and *D_(T)_* respectively. The higher-class cement has a larger specific surface area, so its grains are more fragmented. More packing of the cement grains in the material’s volume indirectly results in a higher apparent density. It is also natural to obtain a higher density of cement paste together with the decrease in *w/b* as a result of increasing the amount of cement at the expense of water. In contrast, the use of MT as a substitute for a part of cement resulted in a slight reduction of *D* by an average of 1.6% and 1.7% for *D_(R)_* and *D_(T)_* respectively; MT has a lower specific density than cement.

The most susceptible parameter to change as a result of the thermal shock is *f_cf_*, for which the average decrease in value was equal to 61.0%. However, the average decrease in *f_c_* was 41.5%. The result of a sudden heating at 250 °C is an increase in the water vapor pressure, which has a destructive effect. The pressure of the saturated water vapor causes the local tensile strength of the material to be exceeded, which results in the propagation of the technological cracks and the occurrence of a distributed damage in the whole material’s volume. In the aspect of stretching the cement matrix, the cracks present are at the same time a break in the continuity of the material’s structure, therefore the *f_cf_* of the cracked sample is very reduced (in this case, cracking due to the thermal load) in relation to the initial value. However, this mechanism looks different in the case of compression of the cement matrix. The surfaces of the separation between cracks in the case of cementitious materials are usually characterized by a high roughness and unevenness [40,41]. As in the case of stretching, in the crack’s area, the material’s cohesion is almost zero; however, the difference is that in the case of stretching, the separation surfaces move away from each other, and in the case of compression they approach each other. At the moment that the contact of both opposite surfaces of the crack occurs, they overlap with each other due to their rough morphology. Direct contact between two surfaces that do not have a structural tie creates a resistance force in the form of friction, which temporarily increases the local cohesion of the material. At some critical value of the compressive stress, the resistance force caused by the friction is exceeded and a slip occurs between the crack’s surfaces, which causes further degradation of the material. The described mechanism results that during compression of the cracked cement matrix, there will not be such a large decrease in *f_c_* as in the case of *f_cf_*. The occurrence of this mechanism is also confirmed by the results obtained by other researchers [42,43,44,45]. Evaporation of free water due to the heating resulted in a decrease in the sample mass. This translates directly into the decrease in *D* of the modified cement pastes, which was on average 10.9%.

### 3.2. Geometric Characteristics of Cluster Cracks

**Figure 6:**
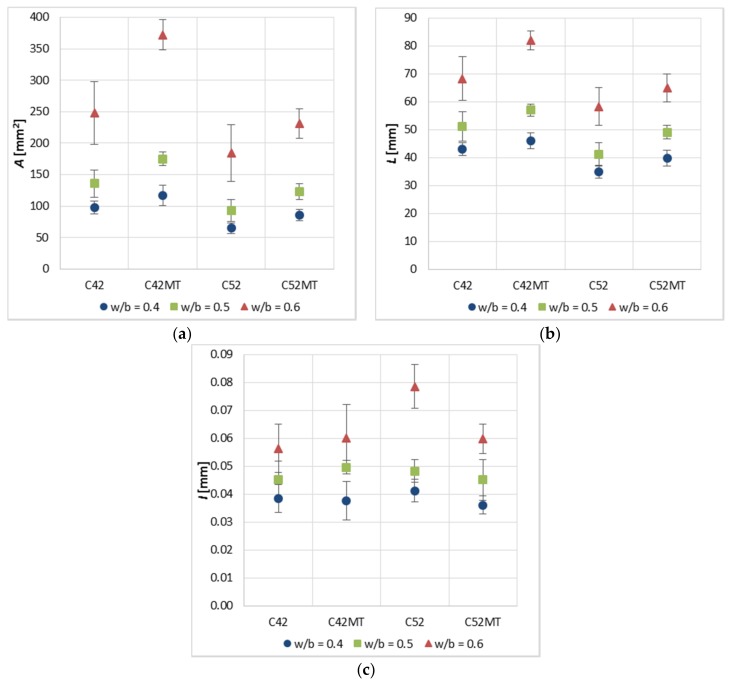
Results of the image analysis of the cement paste surface (error bars mean standard deviations): (**a**) the cluster average area—$\bar{A}$; (**b**) the cluster average perimeter—$\bar{L}$; (**c**) the crack average width—$\bar{I}$.

Figure 6 shows the values measured of the stereological parameters ($\bar{A}$, $\bar{L}$, $\bar{I}$) of the thermal cracks. The obtained results indicate that the geometry of the clusters is dependent on technological variables in the production process of modified cement pastes, i.e., the concentration of the dispersive phase (*w/b*), the cement’s class, and the presence of a pozzolanic additive.

Cement pastes made of a higher-class cement (CEM I 52.5R) were characterized by lower $\bar{A}$ and $\bar{L}$ values by an average of 30.9% and 16.9% from samples made of CEM I 42.5R. However, the inverse relationship was observed in the case of $\bar{I}$; samples of the C52 and C52MT series obtained $\bar{I}$ values by an average higher of 6.5% as compared to C42 and C42MT. The results indicate that the use of a higher-class cement (in this case with a higher fineness) results in a denser network of cluster cracks, increasing the crack width between the clusters at the same time. It was also observed that in each case, along with the decrease in the concentration level of the dispersive phase (increase in *w/b*), the values of the stereological parameters increase.

The use of MT resulted in an increase of $\bar{A}$ by an average of 33.1% and 30.5% compared to the classical cement paste samples (C42 and C42MT)—respectively for samples made of CEM I 42.5R and CEM I 52.5R. In the case of $\bar{L}$ the similar direction of changes as above was observed; samples of the C42MT series reached higher $\bar{L}$ values by an average of 12.8% than samples of the C42 series, and samples of the C52MT series—14.9% from the C52. Both stereological parameters indicate the number of cracks on the surface of the cement matrix. When using MT to modify cement paste, the change in the number of cracks is advantageous, because the higher $\bar{A}$ and $\bar{L}$ values the smaller number of cracks on the material’s surface.

In the case of $\bar{I}$, the effect of MT depends on the cement’s class. For C42MT, the $\bar{I}$ values increased by an average of 4.7% compared to C42. However, for the C52MT an inverse relationship was observed—the $\bar{I}$ value was lower by an average of 14.3% compared to C52. In the case of cement paste made of CEM I 52.5R the beneficial effect of MT was recorded, because the average crack width has been reduced, increasing at the same time the tightness and cohesion of the material.

**Figure 7:**
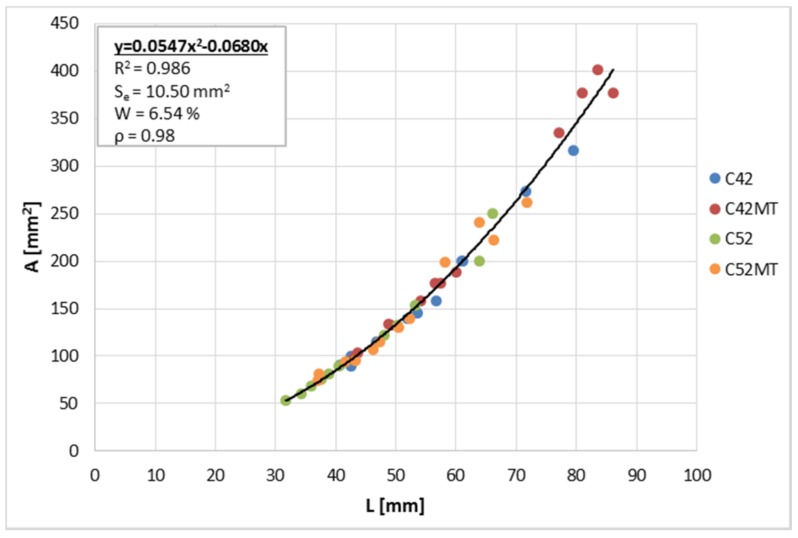
The average cluster area $\bar{A}$ in function of the average cluster perimeter $\bar{L}$; R^2^—the coefficient of determination; *S_e_*—the standard error of estimation; *W*—the random variation coefficient; *ρ*—the correlation coefficient.

The relationship between $\bar{A}$ and $\bar{L}$ is shown in Figure 7. The correlation coefficient between these variables takes a very high value of 0.98. Observing the distribution of points on the graph, it can be concluded that the geometric dependence of the clusters analyzed is strictly defined and invariable, regardless of the technological parameters of the material. The curve equation was calculated using the least squares method (LSM). The values of the diagnostic statistics (R^2^, *S_e_*, *W*) indicate a very good fit of the curve to the empirical data, and an attempt to estimate $\bar{A}$ based on $\bar{L}$ would be very accurate.

**Figure 8:**
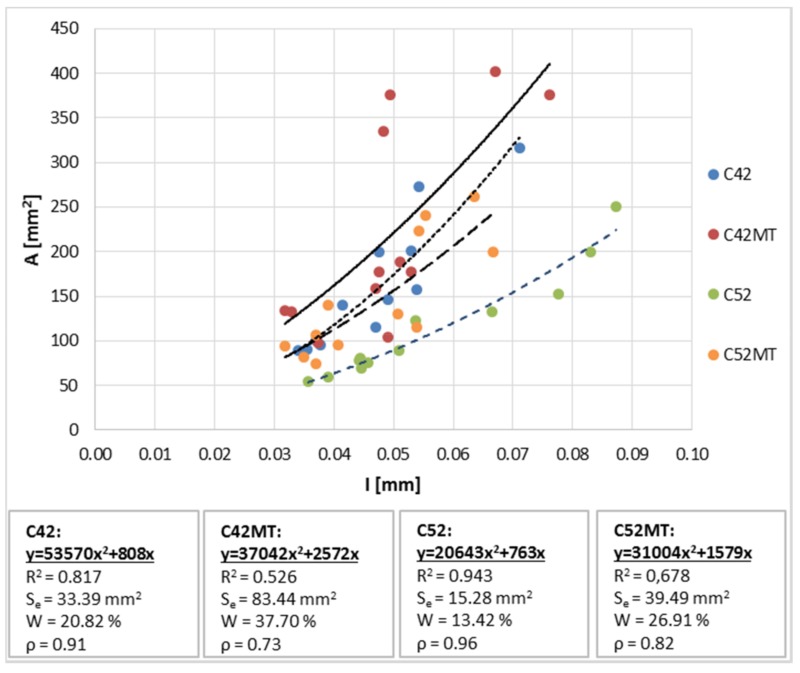
The average cluster area $\bar{A}$ in function of the crack average width $\bar{I}$.

Due to the close relationship between $\bar{A}$ and $\bar{L}$, the relations with $\bar{I}$ were limited to the description with only one parameter of the R^(2)^ space—$\bar{A}$ in this case. The global correlation coefficient between $\bar{A}$ and $\bar{I}$ (Figure 8) is equal to 0.61. It was noticed that with a much greater accuracy the $\bar{A}\left(\bar{I}\right)$ dependence can be described in the criterion related to the division of results depending on the series. In such case the correlation coefficients take higher values ranging from 0.73 to 0.96. As in the case of the $\bar{A}\left(\bar{L}\right)$, the equations of the curves were calculated using the LSM. The best fit of the curve to the empirical data was observed for the C42 and C52 series (the proposed model covers 81.7% and 94.3% of obtained data respectively). The introduction of MT into the structure reduces the diagnostics statistics (e.g., *W* adopts high values equal to 37.7% and 26.9%), which may result in a large error in the case of the $\bar{A}$ estimation based on $\bar{I}$ for the C42MT and C52MT series.

**Figure 9:**
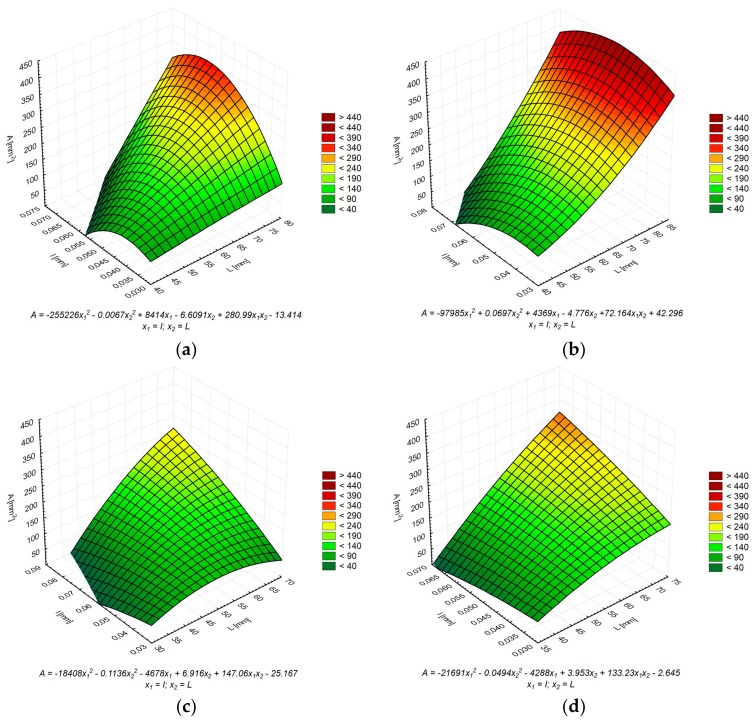
The dependence of the average cluster area $\bar{A}$ on the other stereological parameters ($\bar{L}$ and $\bar{I}$ ) for each series: (**a**) C42; (**b**) C42MT; (**c**) C52; (**d**) C52MT.

To better understand the relationship between the geometric properties of thermal cracks, two-dimensional functional models have been proposed (Figure 9), in which $\bar{A}$ depends on $\bar{L}$ and $\bar{I}$ simultaneously. The second-level model functions were calculated using the LSM in the Statistica software. From the point of view of cohesion, durability and tightness of the cement matrix, the most favorable situation is when the material has a low degree of cracking (high values of $\bar{A}$ and $\bar{L}$), with the smallest crack width at the same time (low $\bar{I}$ values). In this respect, more favorable results were obtained for samples made of CEM I 42.5R, especially for the C42MT series, in which MT was used.

The chemical composition of both cements used is very similar, so in the case analyzed it does not affect the degree of surface cracking. Diversity in the geometric characteristics of clusters in this aspect is conditioned by the fineness of the cement grains, and hence their size.

At the initial stage of the organization of the cement-water structure, between cement grains there are mainly van der Waals forces and capillary forces. During the fixation of clusters at the higher levels of the structure, the capillary forces begin to dominate. The larger the cement grains (CEM I 42.5R), the greater the capillary forces, which cause their stronger attraction to each other. The result is the nucleation of larger binding aggregates due to the presence of larger cement grains, or because of the possibility of cluster fixation consisting of an increased number of grains. The size of the capillary forces, the distribution and concentration of the dispersive phase in the dispersing medium are the factors, which depend on how large the cluster is formed, and how much grains it will consist of. Because of the hydration process, the particles forming the binding aggregate are further tightened, which is related to the occurrence of the volume diffusion and the development of connections between the particles.

Values of the $\bar{A}$, $\bar{L}$, and $\bar{I}$ increase with the increase in the *w/b* ratio. The increase in the distribution degree of cement grains in the space analyzed results in a more even distribution of the capillary forces and intermolecular interactions between the grains. Grains at the initial stage of the structure organization move over greater distances to fix the binding aggregate than they would in the case of a higher concentration of the dispersive phase. Thus, the distances between cluster centers increase, and in the separation surface between clusters connections of reduced strength are formed. This results in a decrease in the resultant cohesion of the material and in the strength parameters of the cement matrix. The MT additive seals the structure of the cement paste. The pozzolanic reaction products can densify the contact area between the clusters, which through additional bonding of the material’s structure limits the increase in the distances between the separation surfaces between clusters. It is particularly visible in the case of samples made of CEM I 52.5R.

### 3.3. Correlations Between Parameters

Table 2 shows a matrix with the Pearson’s correlation coefficients (*r*) between the parameters investigated of the cement pastes. It is worth noting that almost all parameters are very strongly (|r| > 0.9) or strongly (|r| > 0.7) correlated. A change in the parameters tested results in a closely linked change in the remaining characteristics of the cementitious material. This knowledge is useful for estimation purposes. Only the dependence of the tensile strength measured after the temperature load (*f_cf(T)_*) with the other parameters is moderately (|r| < 0.7) or weak correlated (|r| < 0.4), and this parameter is not suitable for proper estimation of other properties both before and after the impact of the thermal shock. In the case of the physico-mechanical properties, the worst correlation is in the case of *f_cf(T)_* and *f_c(T)_*.

Analyzing the strength of correlation of parameters obtained from the image analysis with the physico-mechanical properties of the material, it should be stated that the existing relationships are inverse; in most cases a strong correlation prevails (|r| > 0.7). The highest correlation occurs between the cluster average perimeter and *f_c(T)_* (*r* = −0.86), *D_(R)_* (*r* = −0.90), and *D_(T)_* (*r* = −0.87). Using these quantities, the $\bar{L}$ can be estimated with great accuracy and, looking at the correlation strength between $\bar{A}$ and $\bar{L}$ (*r* = 0.98), also the cluster average area. As in the case of the physico-mechanical properties, the weakest correlations are characterized by the dependences between the R^(2)^ parameters ($\bar{A}$, $\bar{L}$) and *f_cf(T)_*. In the case of $\bar{I}$ and *f_cf(T)_* the correlation is moderate (*r* = 0.63); however the estimation attempt would still be subjected to a large error.

### 3.4. Analysis of the Local Microstructure

Analysis of images obtained with the use of SEM has allowed the identification of thermal microcracks and analysis of the material’s morphology. Below selected SEM photos of the cement paste microstructure made of CEM I 52.5R after thermal interaction are shown. The similar morphology of the microstructure was also observed in the case of CEM I 42.5R cement paste.

**Figure 10:**
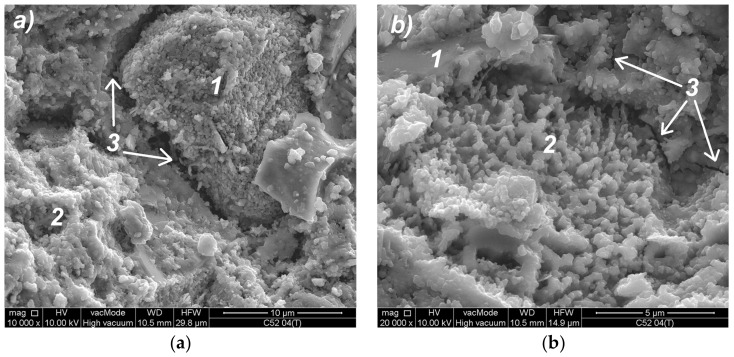
SEM photos of the C52 cement paste microstructure: (**a**) magnification 10,000×; (**b**) magnification 20,000×—another area; descriptions of the indications in the text.

Figure 10a shows the microstructure of a classic cement paste—C52 series. The CSH phase is shown in the form of small isometric grains of hydrated calcium silicates (1) and as a congested gel (2), which corresponds to the type III and IV phases of the CSH according to the classification proposed by Diamond [46]. In the middle of the picture there is a clear crack (3) with residual bridges formed from the CSH phase. In the upper part of Figure 10b, a portlandite plate (1) can be observed with hydrated calcium silicates. In the middle of the image (2) there is a CSH phase at borderline of II (the honeycomb CSH phase) and III Diamond’s type. A microcrack (3) passing through the CSH gel is also visible. The cracks in the microstructure of the cement paste observed are the areas of the separation surfaces between clusters at lower levels, as compared to the clusters observed after the sample’s surface was scanned.

**Figure 11:**
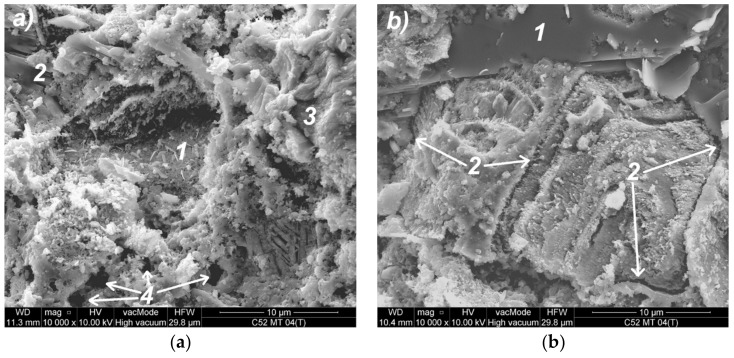
SEM photos of the C52MT cement paste microstructure: (**a**) magnification 10,000×; (**b**) magnification 10,000×—another area; descriptions of the indications in the text.

In the photo of the cement paste with MT (Figure 11a), the residual amount of the tobermorite crystals grown into the CSH phase (1) were observed, and the areas with increased content of calcium hydroxide (2). Mainly the amorphous CSH gel of the type IV (3) dominates, which consists of hydrated silicates and calcium alumino-silicates; the capillaries are clearly visible in the local structure (4). The portlandite plates are clearly visible in the C52MT cement paste microstructure (Figure 11b); they are partially overgrown with the CSH phase. In addition, it is possible to notice isolated areas of the amorphous CSH phase forming clusters at the level of the microstructure, which are separated from each other by microcracks (2).

**Figure 12:**
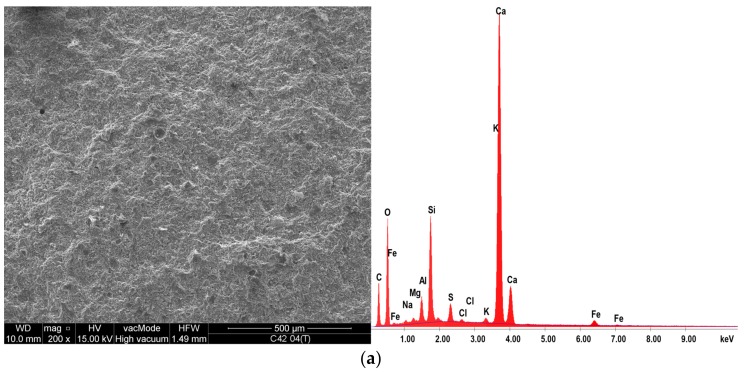

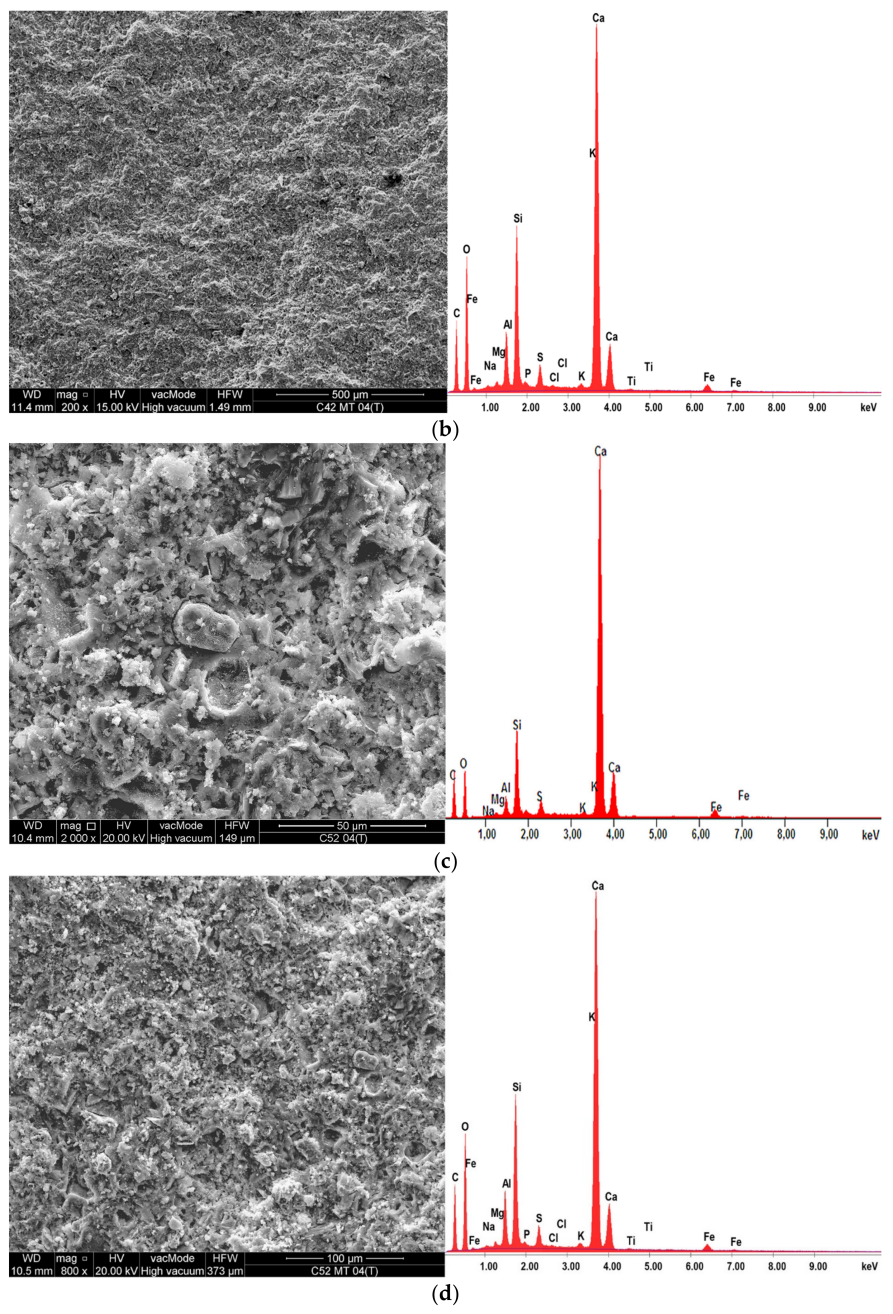
The EDS spectra of the samples tested after thermal action together with the SEM image of the area analyzed: (**a**) C42; (**b**) C42MT; (**c**) C52; (**d**) C52MT.

The EDS X-ray microanalysis results are shown in Table 3, and an exemplary EDS spectrum along with the area analyzed for samples after thermal load is shown in Figure 12. The SEM and EDS analysis confirmed that cement paste subjected to a thermal load at 250 °C is chemically stable; the differences between the content of oxides before and after the thermal action are very small. Both cements are characterized by a very similar chemical composition (Table 1), which resulted in the lack of a clear differentiation in the composition of the hardened cement matrix.

The hydrated calcium silicates present in different morphological forms are the main component of the hardened cement paste (the content of the CSH phase in the 28-day cement paste may exceed 70–80%). Metakaolinite consists mainly of reactive silica (SiO_2_) and aluminum oxide (Al_2_O_5_), which reacts with calcium hydroxide to form hydrated calcium silicates and hydrated calcium alumino-silicates. The EDS analysis showed the MT effect—the Al_2_O_5_ content was greater by an average of 57.7% compared to the C42 and C52 series, and the SiO_2_ content was higher by an average of 6.0%. Indirectly, the reduction of Ca(OH)_2_ content is demonstrated by a reduced amount of CaO—less by 7.9% compared to C42 and C52. The higher amount of the alumina hydrates in the case of a matrix containing MT results in a higher values of an early and normal strength, which is confirmed by other researchers [47,48,49]. The microstructural changes of the MT cement matrix indicated above are responsible for more favorable mechanical properties compared to the classical cement paste.

## 4. Conclusions

The paper presents the results of investigations of the cluster crack structure of cement pastes modified with the metakaolinite. On the basis of analyzed and interpreted results, final conclusions were formulated:

- The use of MT as a substitute for 10% of the cement’s mass positively affects the strength parameters of the reference cement paste. After the thermal shock effect, the beneficial effect of MT was still observed; however, it was smaller compared to the reference samples.
- Computer image analysis can be successfully applied to the quantitative description of the surface structure of cluster cracks.
- Geometric characteristics of the thermal cracks depend on technological variables in the process of cement paste production, i.e., the cement’s class, the *w/b* ratio, the presence of a pozzolanic additive. The process of the structure self-assembly, which affects the cluster layout on the sample’s surface, is shaped by intermolecular interactions in the dispersion environment of cement paste, and by the physico-chemical changes of the system occurring as a result of the cement hydration process.
- The geometrical dependence of clusters (the relationship between $\bar{A}$ and $\bar{L}$) is constant, regardless of the technological variables of the cement paste.
- The $\bar{A}\left(\bar{I}\right)$ relationship depends on the type of cement and the presence of MT, and for estimation purposes, the relationship should be considered with division into series. As the size of the cluster increases, the crack width also increases.
- In the aspect of the material’s durability and resistance to an aggressive environment, the best situation is when the material has a large surface area of clusters, with the smallest width of the cracks. Among the cement pastes tested, the best features in this aspect have obtained a cement paste made of cement with a larger grain size (CEM I 42.5R), in which MT was used.
- The products of the pozzolanic reaction after adding MT to the cement paste seal the contact zone between the clusters. This results in increased cohesion of the material, which translates into a higher mechanical strength.
- Analysis of the correlation indicated that the stereological parameters of the cracks are strongly correlated with the physical and mechanical properties of the cement pastes. Only in the case of *f_cf(T)_* dependencies with the cracks’ geometry are characterized by a weak correlation and the estimation attempt would be burdened with a large error.
- SEM analysis confirmed that cluster cracks have a fractal character and the structures visible in the macroscale are also found in the microscale.

## Figures and Tables

**Table 1:** Characteristics of the Portland cements used.

Cement’s Class	Chemical Analysis [%]								
	SiO_2_	Fe_2_O_3_	Al_2_O_3_	CaO	MgO	SO_3_	Cl	Na_2_O	K_2_O
CEM I 42.5R	20.18	3.39	4.38	64.79	1.17	2.91	0.083	0.26	0.49
CEM I 52.5R	20.19	3.30	4.33	64.76	1.17	3.16	0.078	0.26	0.48
**Mineral Composition [%]**					**Blaine Specific Surface Area [cm^2^/g]**				
**Cement’s Class**	**C3S**	**C2S**	**C3A**	**C4AF**					
CEM I 42.5R	63.41	8.92	5.88	10.31	4010				
CEM I 52.5R	62.97	9.28	5.90	10.03	4596				

**Table 2:** Correlation matrix of the parameters examined of the cement pastes.

Parameter	*f_c(R)_*	*f_c(T)_*	*f_cf(R)_*	*f_cf(T)_*	*D_(R)_*	*D_(T)_*	$\bar{A}$	$\bar{L}$	$\bar{I}$
***f_c(R)_***	1.00	-	-	-	-	-	-	-	-
***f_c(T)_***	0.98	1.00	-	-	-	-	-	-	-
***f_cf(R)_***	0.95	0.91	1.00	-	-	-	-	-	-
***f_cf(T)_***	0.43	0.33	0.63	1.00	-	-	-	-	-
***D_(R)_***	0.95	0.97	0.94	0.50	1.00	-	-	-	-
***D_(T)_***	0.94	0.94	0.94	0.56	0.99	1.00	-	-	-
$\bar{A}$	−0.76	−0.81	−0.75	−0.39	−0.85	−0.82	1.00	-	-
$\bar{L}$	−0.81	−0.86	−0.79	−0.37	−0.90	−0.87	0.98	1.00	-
$\bar{I}$	−0.79	−0.75	−0.78	−0.63	−0.82	−0.84	0.61	0.68	1.00

**Table 3:** The oxidized composition of the cement pastes before and after the thermal load.

Oxide	Percentage of Oxides before/after Thermal Load [%]							
	C42		C42MT		C52		C52MT	
	before	after	before	after	before	after	before	after
Na_2_O	1.17	0.98	0.78	0.62	1.00	0.79	0.65	0.59
MgO	1.13	1.18	1.09	1.07	1.03	1.06	0.97	0.98
Al_2_O_5_	5.90	6.36	9.58	9.98	6.34	6.58	10.14	10.01
SiO_2_	21.41	21.56	23.01	23.22	22.43	22.87	23.54	23.78
SO_3_	3.91	4.45	4.23	4.35	3.44	3.78	4.00	4.22
K_2_O	1.02	0.69	1.01	0.88	0.95	0.52	0.94	0.74
CaO	62.55	61.68	57.31	56.86	61.80	61.30	56.90	56.68
Fe_2_O_3_	2.91	3.10	2.99	3.02	3.01	3.10	2.86	3.00
